# Sensory integration, postural stability, and quality of life: a multidimensional assessment in adult hearing aid users

**DOI:** 10.1007/s00405-026-10346-1

**Published:** 2026-06-01

**Authors:** Sevgi Kutlu, Zehra Aydogan, Banu Baş, Betül Temir, Cem Meço

**Affiliations:** 1 Faculty of Health Sciences, Department of Audiology, Ankara University, Ankara, Türkiye; 2 Faculty of Health Sciences, Department of Audiology, Ankara Yıldırım Beyazıt University, Ankara, Türkiye; 3 Institute of Health Sciences, Department of Audiology and Speech Disorder, Ankara University, Ankara, Türkiye; 4 Department of Otorhinolaryngology, Faculty of Medicine, Ankara University, Ankara, Türkiye; 5 Department of Otorhinolaryngology-Head and Neck Surgery, Salzburg Paracelsus Medical University, Salzburg, Austria; 6 Department of Otolaryngology, Head and Neck Surgery, Cornell University, Weill Cornell Medical College, New York, NY USA

**Keywords:** Sensory integrity, Balance, Hearing loss, Quality of life, Postural stability, Sensory processing

## Abstract

**Purpose:**

The aim of this study is to multidimensionally evaluate sensory processing profiles, postural stability, and health-related quality of life in adult hearing aid users and to compare the findings with those of normal-hearing individuals.

**Methods:**

A total of 52 adults participated in the study, including 21 hearing aid users (mean age: 47.48 ± 10.55 years) and 31 age- and gender-matched normal-hearing individuals (mean age: 41.94 ± 10.89 years). Sensory processing was assessed using the Adolescent/Adult Sensory Profile, postural stability was evaluated by computerized dynamic posturography, and quality of life was measured using the SF-36 Health Survey. Group comparisons and associations between variables were analyzed using appropriate statistical methods.

**Results:**

Hearing aid users demonstrated significantly lower scores in the Sensory Seeking, Taste/Smell Processing, and Activity Level subscales compared with normalhearing individuals. In the postural stability assessment, composite balance as well as visual and vestibular subsystem scores were significantly lower in the hearing loss group. Significant correlations were observed between selected sensory processing subdimensions and postural stability parameters. Regarding quality of life, hearing aid users showed significantly poorer Social Functioning scores, while the difference in Physical Functioning did not remain significant after multiple-comparison correction.

**Conclusions:**

Hearing loss appears to affect not only auditory function but also sensory processing, postural control, and aspects of quality of life. These findings support the need for a multidimensional approach in the clinical evaluation and rehabilitation of adult hearing aid users.

## Introduction

Globally, hearing loss is one of the leading causes of years lived with disability, ranking third [1]. According to the World Health Organization, more than 5% of the world’s population, approximately 430 million people, require rehabilitation due to hearing loss-related disabilities [2]. Sensorineural hearing loss is the most common type of hearing loss in adults and can be caused by many factors, including aging, genetic predisposition, noise exposure, ototoxic drugs, and chronic diseases [3]. Hearing loss has been reported to be associated with negative effects in many areas, including difficulty understanding speech in noise, social isolation, reduced quality of life, depression, risk of falls, and dementia [4–6].

All information from the environment is received through sensory receptors. The regular and coordinated functioning of sensory systems enables individuals to perceive environmental stimuli more effectively and increases their adaptation to the environment [7]. Sensory integration refers to the process by which the central nervous system processes and organizes different sensory inputs (auditory, visual, tactile, olfactory, gustatory, vestibular, proprioceptive, and interoceptive) and generates appropriate behavioral responses [8]. Impairment in one or more sensory inputs can negatively affect the holistic and functional use of this information in daily life [9]. Insufficient auditory stimulation due to hearing loss limits the quantity and quality of sensory stimuli [10]. It has been suggested that the reduction in auditory input in cases of hearing loss may affect the functioning of other senses through the brain’s cross-modal plasticity mechanisms and that this situation may pave the way for the development of compensatory sensory strategies [11, 12]. In this context, it has been reported that prolonged auditory deprivation triggers significant functional plasticity in the central nervous system, leading to increased involvement of the auditory cortex in processing information from other sensory modalities [13]. The clinical implications of such reorganization processes may manifest as difficulties in sensory processing areas in individuals with severe hearing loss. Indeed, sensory processing difficulties have been reported in individuals with bilateral profound sensorineural hearing loss who use cochlear implants [10]. Analyzing sensory processing profiles may enable the identification of specific sensory difficulties and the creation of individualized treatment plans [14].

Changes in sensory integration processes are thought to play a decisive role in postural control and balance mechanisms [15]. In individuals with hearing loss, vestibular dysfunction may also occur due to the close anatomical relationship between the cochlea and the vestibular system [16, 17]. Studies show that postural instability becomes more pronounced when auditory environmental input is insufficient [18]. Therefore, the objective assessment of postural stability in individuals with hearing loss is clinically important. Postural stability is achieved through the integration of sensory inputs from the vestibular, somatosensory, and visual systems. These multiple sensory inputs are integrated in the central nervous system for the formation of body schema and the planning of postural movements [19].

Hearing and balance problems can lead to social isolation and a decrease in quality of life by making it difficult to process sensory stimuli adequately [10]. This situation can negatively affect individuals’ participation in daily life activities, independence, and psychosocial well-being.

A review of the literature reveals that most studies on individuals with hearing loss focus on a single area, while studies that simultaneously assess sensory integration, postural stability, and quality of life are limited. The primary aim of this study was to compare computerized dynamic posturography (CDP) composite, visual (VIS), and vestibular (VEST) scores between adult hearing aid users and individuals with normal hearing. Secondary aims included assessing whether hearing loss is associated with altered sensory processing patterns (using the Adolescent/Adult Sensory Profile) and reduced health-related quality of life (using the SF-36), and exploring the correlations between these multidimensional outcomes. Based on previous literature and our 2024 cochlear implant study, we hypothesized that hearing aid users would demonstrate altered CDP scores compared to controls, reflecting a potential association between chronic auditory deprivation and multisensory postural control. Additionally, poorer postural stability was expected to be associated with altered sensory processing patterns and reduced quality of life, particularly in social and physical functioning domains.

## Materials and methods

This study was approved by the Ankara University Faculty of Medicine Human Research Ethics Committee, decision number and code İ07-635-25 (approval date: 14 August 2025). The study was conducted in accordance with the principles of the Declaration of Helsinki. Written informed consent was obtained from all participants, including hearing aid userers and control subjects. Who expressed their willingness to participate in this study.

### Participants

Participant recruitment began after ethical approval in August 2025 and was conducted at the Audiology, Speech and Balance Unit of Ankara University. Participants were recruited on a voluntary basis using a convenience sampling method. The study group included 21 individuals (11 females and 10 males) with bilateral sensorineural hearing loss, bilateral behind-the-ear hearing-aid use, no balance complaints, and no physical disabilities that could cause balance problems, with a mean age of 47.48±10.55 years. All participants were regular bilateral hearing-aid users, and the duration of hearing-aid use ranged from 6 months to 15 years. Individuals with single-sided deafness and asymmetric hearing loss were excluded from the study. Individuals with known vestibular disorders (e.g., benign paroxysmal positional vertigo, vestibulopathy, Ménière’s disease), neurological diseases affecting balance, uncontrolled diabetes, peripheral neuropathy, history of otologic surgery (except hearing-aid use), or use of medications that could influence vestibular function or postural stability (e.g., vestibular suppressants, sedatives) were excluded from the study. Participants with polypharmacy that could affect balance or sensory processing were also not included.

The control group included 31 individuals (19 females and 12 males) with bilateral normal hearing and no history of balance disorders, with a mean age of 41.94±10.89 years.

None of the participants or data from our previous 2024 cochlear implant study were reused in the present study. Participant recruitment fort his current study started after ethical approval in August 2025, and all participants in this study were hearing-aid users, whereas the previous study included only cochlear implant users. Participants in the cochlear implant study were recruited between March 2023 and June 2023, and therefore the two datasets were completely independent in terms of both participants and data collection periods.

### Assesment tools

#### Pure tone audiometric testing

Hearing sensitivity was evaluated in a sound-treated environment using a GSI AUDIOSTAR PRO audiometer (Minnesota, USA) with TDH-39 supra-aural headphones. Air-conduction thresholds were obtained across octave and inter-octave frequencies ranging from 125 to 8000 Hz. Pure-tone averages were calculated separately for each ear by averaging thresholds at 0.5, 1, 2, and 4 kHz. Hearing levels with a pure-tone average below 25 dB HL were classified as normal hearing [20].

#### Adolescent/Adult sensory Profile

The Adolescent/Adult Sensory Profile is a test consisting of 60 questions related to 6 sensory processing categories (taste/smell, movement, vision, touch, auditory development, activity level) that has been in use since 2002. The validated Turkish version of the scale was used in the present study [21]. The scale was developed for use in adolescents and adults aged 11 and older and is evaluated based on normative data specific to three separate age groups (11–18 years, 18–65 years, and ≥65 years). Based on Dunn’s sensory processing theory, the scale includes four quadrants: sensory sensitivity, sensory avoiding, low registration, and sensory seeking. In each subscale, individuals are rated according to their responses to sensory experiences using the options “much more than most people,” “more than most people,” “similar to most people,” “less than most people,” and “much less than most people” and scores are interpreted according to normative classification ranges provided in the test manual. Higher or lower scores indicate deviations from typical sensory processing patterns depending on the quadrant. Sensory sensitivity is characterized by an individual having a low threshold for sensory stimuli and responding to these stimuli more intensely than normal. Sensory avoidance refers to an individual finding sensory stimuli unpleasant and consciously avoiding them. Under-registration describes a situation where an individual shows less awareness of sensory inputs than normal or responds to these inputs more slowly. Sensory seeking, on the other hand, is related to an individual deriving pleasure from sensory inputs and actively seeking out such stimuli. For example, lower Sensation Seeking scores indicate reduced active engagement with sensory input, whereas higher scores in Sensory Sensitivity and Sensory Avoiding reflect increased sensory reactivity [22].

#### Computerized dynamic posturography (CDP)

The posturographic test was performed using the Equitest posturograph manufactured by NeuroCom International^®^ [23]. Computerized dynamic posturography, a valuable tool in investigating sensory, motor, and central adaptive disorders, provides an objective measurement of postural control [24]. Computerized Dynamic Posturography is a method that quantitatively assesses the contribution of the vestibular, visual, and somatosensory systems to postural control under different sensory conditions that simulate daily life conditions [25]. The test system consists of a moving platform on which the individual stands and a moving visual cabin surrounding the platform. The assessment is performed under six different sensory stimulation conditions in which visual stimuli are manipulated and the foot support surface or visual environment is moved. Through these conditions, specific components of the balance system are selectively suppressed, and the relative contributions of the sensory systems affecting postural control can be analyzed based on the results obtained. The Composite Score (COMP) calculated during the Sensory Organization Test reflects the weighted average of the scores obtained from the six test conditions and reveals the individual’s dependence on somatosensory (SOM), visual (VIS), and vestibular (VES) inputs while maintaining balance and the visual preference (PREF) index, which indicates the extent to which the individual relies on this information, independent of the accuracy of visual information. Test results were interpreted using age-referenced normative data provided by the manufacturer. For the Composite (COMP) score, the lower normative limits were 70 (20–59 years), 68 (60–69 years), and 64 (70–79 years). Scores below these thresholds reflect reduced effectiveness of multisensory integration for maintaining postural stability, and group differences were interpreted in relation to these normative benchmarks [25, 26].

#### Short Form-36 (SF-36)

The SF-36 Health Survey is a 36-item standard health status assessment tool derived from the large-scale pool created within the RAND Medical Outcomes Study (MOS) [27]. This 36-item scale is used to assess an individual’s health-related quality of life through eight subscales: physical functioning, role limitations due to physical health, Role limitations due to emotional problems, Energy/fatigue, emotional well-being, social functioning, pain, and general health perception. The scale is a self-report measure that can be completed by the individual in a very short time. SF-36 scores were transformed to a standardized 0–100 scale, with higher scores indicating better health status [28]. In the present study, the validated Turkish version of the SF-36 Health Survey was utilized [29].

### Statistical analysis

Statistical analysis of the data was performed using the SPSS 26.0 (IBM Corp., Armonk, NY, USA) software package. The normality of variables was examined using visual (histogram and Q-Q plots) and analytical methods (Shapiro-Wilk test). Descriptive Statistics: Mean and standard deviation (Xˉ±SS) were used for numerical data, and frequency (n) and percentage (%) values were used for categorical data. The Independent Samples T-test was applied for the intergroup comparison of posturography (SOT) and Sensory Profile data showing a normal distribution. For the SF-36 subdomains, the choice of statistical test depended on distributional properties: normally distributed variables were analyzed using the Independent Samples t-test, whereas non-normally distributed variables were analyzed using the Mann–Whitney U test. SF-36 domain scores were transformed to a standardized 0–100 scale, with higher scores indicating better health status. The Spearman Correlation Coefficient (ρ) was calculated to determine the direction and strength of the relationship between sensory profile questionnaire scores and objective posturography parameters. A one-way ANCOVA was applied to control for the possible effect of age on balance performance and to confirm the independence of age from the difference between groups. The difference in gender distribution between the groups was evaluated using the Pearson Chi-Square test. Given the large number of parallel tests performed across the Adolescent/Adult Sensory Profile (AASP) subscales, CDP/SOT parameters, SF-36 domains, and correlation analyses, the false discovery rate (FDR) was controlled using the Benjamini–Hochberg procedure within each family of related outcomes. Adjusted p-values (q-values) were calculated, and statistical significance after correction was accepted at q<0.05. The statistical significance level was set at *p*<.05 for all analyses. Effect sizes were calculated using Hedges’ g to estimate the magnitude of between-group differences, and 95% confidence intervals (95% CI) were reported to reflect the precision of these estimates.

The primary outcome of the study was the CDP Composite score (COMP). Secondary outcomes included VIS and VEST ratios and selected SF-36 domains (Physical and Social Functioning), which were defined a priori based on study hypotheses and previous literature. All AASP subscales were analyzed to comprehensively evaluate sensory processing patterns; however, particular attention was given to Sensation Seeking, Taste/Smell Processing, and Activity Level due to their theoretical and empirical relevance to postural control.

An a priori power analysis was conducted using G*Power (version 3.1) to determine the required sample size for the primary comparison between two independent groups. The assumption of a large effect size (Cohen’s d = 1.02) was based on previous findings in similar clinical populations, specifically adult cochlear implant users, where substantial differences in multisensory integration and postural stability (CDP Composite scores) were observed compared to normal-hearing controls. Given the primary outcome of the present study, this threshold was deemed appropriate to detect clinically meaningful differences in postural control. With a significance level of α = 0.05 and a statistical power of 0.85, the minimum required sample size was calculated as 38 participants (19 per group). The final sample size (*n* = 52) exceeded this requirement, ensuring that the study was adequately powered even if the actual effect size was slightly more moderate than initially estimated.

## Results

The analyses revealed no statistically significant differences between the groups in terms of age (*p*=.075) and gender distribution (*p*=.523). In contrast, when pure tone audiometry (PTA) results were examined, statistically significant differences were observed between groups in terms of both right ear (*p* < .001) and left ear (*p* < .001) PTA averages (Table 1).

**Table 1:** Demographic and audiological characteristics of the groups

	Control Group (*n*=31)	Study Group (*n*=21)	*p*
Age (years, Mean ± SD)	41.94±10.89	47.48±10.55	0.075
Gender (Female/Male)	19/12	11/10	0.523
PTA -Right ear (dB HL, Mean ± SD)	4.03 ±3.73	50.38 ±18.66	*0.001
PTA -Left ear (dB HL, Mean ± SD)	3.71 ±3.52	53.48 ±23.34	*0.001
PTA-Better ear (dB HL, Mean ± SD)	3.09 ±3.32	41.10 ±16.78	*0.001
PTA- Worse ear (dB HL, Mean ± SD)	4.16 ±3.93	53.52 ± 17.58	*0.001

*PTA* Pure-tone average, *SD* Standard deviation, *dB HL* decibel hearing level. *= *p*<.001

The analysis revealed that individuals with hearing loss had statistically significantly lower scores on the Sensory Seeking (*p* = .009), Taste/Smell Processing (*p* = .005), and Activity Level (*p* = .020) subscales compared to individuals with normal hearing (*p* < .05). Effect size analysis indicated a moderate-to-large difference for Sensation Seeking (g = 0.73, 95% CI: 0.16 to 1.30), a large difference for Taste/Smell Processing (g = 0.84, 95% CI: 0.26 to 1.42), and a moderate difference for Activity Level (g = 0.69, 95% CI: 0.10 to 1.28). In contrast, no significant differences were found between the groups in the subdimensions of Low Registration, Sensory Sensitivity, Sensory Avoidance, Motor Processing, Visual Processing, Tactile Processing, and Auditory Processing (*p* > .05), and effect sizes for these comparisons were small or negligible (Table 2).

**Table 2:** Intergroup comparison of sensory profile subdimensions

Subdimensions	Control group (Mean±SD)	Study group (Mean±SD)	Mean difference (95%CI)	*p*	Effect size g (95% CI)
Low Registration	32.52 ± 5.20	35.65 ± 7.84	−3.13 (−6.80 to 0.53)	0.092	−0.47 (−1.02 to 0.08)
Sensation Seeking	44.35 ± 5.29	38.85 ± 9.08	5.50 (1.47 to 9.55)	*0.009	0.73 (0.16 to 1.30)
Sensory Sensitivity	38.97 ± 7.64	39.50 ± 7.03	−0.53 (−4.80 to 3.74)	0.803	−0.07 (−0.62 to 0.48)
Sensation Avoiding	38.48 ± 7.56	39.05 ± 7.64	−0.57 (−4.94 to 3.81)	0.796	−0.08 (−0.63 to 0.47)
Taste/Smell Processing	24.74 ± 4.21	20.90 ± 4.94	3.84 (1.24 to 6.44)	*0.005	0.84 (0.26 to 1.42)
Movement Processing	19.39 ± 3.59	19.70 ± 4.42	−0.31 (−2.58 to 1.96)	0.783	−0.06 (−0.61 to 0.49)
Visual Processing	27.52 ± 4.86	24.95 ± 5.68	2.57 (−0.43 to 5.56)	0.091	0.50 (−0.05 to 1.05)
Touch Processing	35.10 ± 5.22	32.45 ± 6.77	2.65 (−0.74 to 6.03)	0.122	0.45 (−0.10 to 1.00)
Activity Level	27.10 ± 4.79	23.50 ± 5.81	3.60 (0.59 to 6.60)	*0.020	0.69 (0.10 to 1.28)
Auditory Processing	29.58 ± 6.73	31.45 ± 8.02	−1.87 (−6.05 to 2.32)	0.374	−0.26 (−0.81 to 0.29)

*= *p*<.05, SD=Standart Deviation. Note: One participant in the hearing-aid group had missing data for the Sensory Profile; therefore, analyses for Table 2 were conducted with *n*=31 controls and *n*=20 hearing-aid users

*indicates results that remained statistically significant after Benjamini–Hochberg False Discovery Rate (FDR) correction for multiple comparisons (q = 0.05). SD: Standard Deviation

The differences in posturography performance between the hearing-impaired group and the normal-hearing group were analyzed using the Independent Samples T-Test. According to the analysis results, the COMP (*p*<.001), VIS (*p*<.001), and VEST (*p*=.020) scores of the hearing-impaired group were found to be statistically significantly lower than those of the normal-hearing group. No significant difference was found between the groups in SOM and PREF scores (*p*>.05) (Table 3).

**Table 3:** Comparison of groups based on computerized dynamic posturography scores

	Group	N	Mean	SD	t	p	Mean difference (95% CI)	Effect size g (95% CI)
COMP	Control Group	31	86.13	3.83	4.827	*0.000	6.51 (3.80 to 9.21)	1.40 (0.78 to 2.01)
	Study Group	21	79.62	5.90				
VIS	Control Group	31	94.52	4.12	6.293	*0.000	10.51 (7.16 to 13.87)	1.83 (1.17 to 2.48)
	Study Group	21	84.00	7.86				
VEST	Control Group	31	83.29	7.55	2.403	*0.020	5.86 (0.96 to 10.76)	0.69 (0.12 to 1.27)
	Study Group	21	77.43	10.03				
SOM	Control Group	31	98.97	2.07	0.260	0.796	0.15 (-1.06 to 1.38)	0.07 (-0.47 to 0.62)
	Study Group	21	98.81	2.27				
PREF	Control Group	31	98.87	4.52	1.332	0.189	1.96 (-0.99 to 4.93)	0.38 (-0.17 to 0.93)
	Study Group	21	96.90	6.12				

*COMP* composite score, *SOM* somatosensory, *VIS* visiual, *VEST* vestibular, *PREF* visual preference, *SD* standart deviation, *p < 0,05, *indicates results that remained statistically significant after Benjamini–Hochberg False Discovery Rate (FDR) correction for multiple comparisons (q = 0.05). SD: Standard Deviation

In the one-way analysis of covariance (ANCOVA) conducted to control for the potential confounding effect of age, age was included in the model as a covariate. According to the analysis results: In the Composite score, the effect of age was not statistically significant (F(1,49)=3.289, *p*=.076). However, the group effect was significant (F(1,49)=27.382, *p*<.001, η²*p*=.358). This finding shows that the difference between the groups continues even when age is controlled. In the VIS score, no significant effect of age was observed (F(1,49)=0.246, *p*=.622). The group effect is strong and significant (F(1,49)=38.097, *p*<.001, η²*p*=.437). In the VEST score, the age effect is not significant (F(1,49)=1.733, *p*=.194), but the group effect is significant (F(1,49)=7.143, *p*=.010, η²*p*=.127). In the PREF score, neither the age (F(1,49)=3.501, *p*=.067) nor the group (F(1,49)=3.200, *p*=.080) effects reached statistical significance (Table 4). The findings suggest that age does not show a significant covariate effect on the dependent variables and that the group differences observed in Composite, VIS and VEST scores are not age-related.

**Table 4:** ANCOVA results with age included as covariate

Dependent Variable	Group F(1,49)	*p*	Partial η²	Age F(1,49)	*p*	Partial η²	Model *R*²
Composite	27.382	<0.001	0.358	3.289	0.076	0.063	0.361
VIS	38.097	<0.001	0.437	0.246	0.622	0.005	0.445
VEST	7.143	0.010	0.127	1.733	0.194	0.034	0.134
PREF	3.200	0.080	0.061	3.501	0.067	0.067	0.099

df = 1,49, covariant: age

Individuals with hearing loss were found to have statistically significantly lower scores on the Social Functioning (*p* = .013) and Physical Functioning (*p* = .037) subscales of the SF-36 health-related quality of life scale compared to individuals with normal hearing. Physical Functioning showed a lower score in the hearing loss group at the uncorrected level (*p* = .037); however, this difference did not remain statistically significant after Benjamini–Hochberg FDR correction. In contrast, no significant differences were found between the groups in the subdimensions of Energy/Vitality, Mental Health, General Health Perception, Pain, Physical Role Difficulty, and Emotional Role Difficulty (*p* > .05) (Table 5).

**Table 5:** Comparison of SF-36 quality of life scores among groups

SF-36 Alt Boyutu	Control group (Mean± SD)	Study group (Mean± SD)	Test statisctic	*p*	Effect size g/r (95% CI)
Physical function	29.42 (IQR)	20.70 (IQR)	U=204.0	*.037	**r=0.29 (0.92 to 0.52)**
Role limitations due to physical health	27.50 (IQR)	23.68 (IQR)	U=263.5	.340	r=0.13 (-0.15 to 0.39)
Role limitations due to emotional problems	28.77 (IQR)	21.70 (IQR)	U=224.0	0.076	r=0.24 (-0.03 to 0.48)
Energy/fatigue	51.13±21.78	54.50±21.87	t=−.539	.593	g=-0.15 (-0.70 to 0.40)
Emotional well-being	61.68±17.73	54.40±18.73	t=1.400	.168	g=0.39 (-0.16 to 0.94)
Social functioning	71.37±20.18	55.03±24.78	t=2.581	*.013	**g=0.74 (**0.17 to 1.31)
Pain	68.16±27.00	63.00±26.49	t=0.671	.505	g=0.19 (-0.36 to 0.73)
General health	61.00±21.23	53.75±16.77	t=1.288	.204	g=0.36 (-0.19 to 0.91)

*p < 0,05, *IQR* interquartile range, *U* Mann–Whitney U test. *indicates results that remained statistically significant after Benjamini–Hochberg False Discovery Rate (FDR) correction for multiple comparisons (q = 0.05). SD: Standard Deviation

To account for multiple comparisons across the Sensory Profile subscales, CDP/SOT parameters, and SF-36 domains, the Benjamini–Hochberg false discovery rate (FDR) correction was applied (q=0.05). After correction, the differences observed in Composite, VIS, VEST, Taste/Smell Processing, Sensory Seeking, Activity Level, and Social Functioning remained statistically significant. However, the difference in the Physical Functioning domain did not remain significant after FDR adjustment (uncorrected *p*=.037) and should therefore be interpreted with caution (Table 6)

**Table 6:** Results of the Benjamini–Hochberg False Discovery Rate (FDR) correction for multiple comparisons (q = 0.05)

Tested variable	Raw *p*-value	Rank (i)	Critical value (i/12) × 0.05	Result
Composite (Posturography)	0.0001*	1	0.0042	**Significant**
VIS (Posturography)	0.0001*	2	0.0083	**Significant**
Taste/Smell Processing (Sensory)	0.005	3	0.0125	**Significant**
Sensory Seeking (Sensory)	0.009	4	0.0167	**Significant**
Social Functioning (SF-36)	0.013	5	0.0208	**Significant**
VEST (Posturography)	0.020	6	0.0250	**Significant**
Activity Level (Sensory)	0.020	7	0.0292	**Significant**
Physical Functioning (SF-36)	0.037	8	0.0333	Not statistically significant after FDR correction

*Remained significant following Benjamini–Hochberg adjustment

To examine the relationship between the subdimensions of the Sensory Profile Questionnaire used in the study and the BDP (SOT) parameters, Spearman correlation analysis was applied, and the results are presented in Table 7. The analysis revealed a positive and moderately significant relationship between Sensory Seeking and the VEST score (ρ = 0.382, *p* = .006). Furthermore, a positive and significant correlation was found between Sensory Seeking and the VIS score (ρ = 0.280, *p* = .047). The Activity Level subscale showed positive and significant relationships with both the VEST (ρ = 0.294, *p* = .036) and PREF (ρ = 0.313, *p* = .026) scores. In contrast, a negative significant relationship was found between the Touch Processing subscale and the SOM score (ρ = −0.302, *p* = .031). These findings indicate that there are significant relationships between sensory processing characteristics and postural control systems.

**Table 7:** Correlation analysis results between sensory profile and posturography scores

Variables	*N*	ρ (Spearman’s rho)	*p*
Sensation Seeking - VEST	52	0.382 [0.117 to 0.595]	**0.006
Touch Processing -SOM	52	−0.302 [−0.535 to −0.030]	*0.031
Activity Level - VEST	52	0.294 [0.022 to 0.526]	*0.036
Activity Level - PREF	52	0.313 [0.043 to 0.541]	*0.026
Sensation Seeking - VIS	52	0.280 [0.007 to 0.514]	*0.047

*p < .05, **p < .01 (two-tailed); *N* sample size, *ρ* Correlation Coefficient, *SOM* somatosensory, *VIS* visual, *VEST *vestibular, *PREF* preference score. ρ represents Spearman’s correlation coefficient. 95% CI: 95% Confidence Interval for the correlation coefficient. * indicates significance maintained after Benjamini–Hochberg FDR correction

## Discussion

In this study, sensory processing profiles, postural stability, and quality of life were assessed multidimensionally in adult hearing aid users, and the findings were compared with those of individuals with normal hearing. The findings indicate that there are significant differences in sensory processing areas among hearing aid users, that postural stability is particularly affected under certain sensory conditions, and that there is a decrease in some subdimensions of quality of life. These results suggest that hearing loss is associated with differences not only in auditory perception but also an individual’s daily functioning and perceived quality of life through sensory integration processes and postural control mechanisms. The study’s findings demonstrate that addressing the sensory and postural changes accompanying hearing loss together is clinically meaningful.

The way sensory inputs are processed forms the basis of learning in early development and contributes to the formation of adaptive behavior patterns in adulthood. The building block of behavior is the integration and interpretation of sensory information from the organism and the environment [30]. Insufficient auditory stimulation due to hearing loss limits the quantity and quality of sensory stimuli [10]. Degenerative changes in the brain and sensory systems associated with sensorineural hearing loss of various etiologies can negatively affect the processing of sensory information and responses to this information [14]. Our study found that individuals with hearing loss had statistically significantly lower scores on the Sensation Seeking, Taste/Smell Processing, and Activity Level subscales compared to individuals with normal hearing. The lower scores of individuals with hearing loss on these subscales suggest that these individuals may have more limited active exploration behaviors and overall arousal levels in response to environmental stimuli compared to individuals with normal hearing. In particular, the decrease in sensory seeking indicates that the individual’s motivation to increase environmental sensory input may be low. Over time, this situation may lead the individual to adopt a more passive sensory strategy. Central sensory integration changes accompanying hearing loss may also cause processing differences in non-auditory senses [31]. Considering the role of chemical senses such as taste and smell in multisensory integration, a decrease in auditory input may indirectly affect the functional use of these systems. In a study conducted with cochlear implant users, activity levels were found to be lower than in the group with normal hearing, similar to our study [10]. Reduced auditory input may limit sensory feedback from environmental interactions, thereby decreasing an individual’s sensation seeking behavior [10]. Lower activity levels may be related to decreased environmental engagement, reduced energy levels, or avoidance behaviors frequently reported in individuals with hearing loss. This situation can be evaluated in connection with both a decrease in sensation seeking and a limitation in postural and motor participation. In our study, the postural stability of individuals with hearing loss was evaluated using computerized dynamic posturography. According to the analysis results, the COMP, VIS, and VEST scores of the group with hearing loss were found to be statistically significantly lower than those of the group with normal hearing. These findings suggest a correlation between hearing loss and lower performance in the multisensory components of postural control. In particular, the low composite score may reflect diminished effectiveness of sensory integration, potentially influenced by unmeasured subclinical vestibular dysfunction [10]. Performance deficits detected in the visual and vestibular subsystems may be related to vestibular dysfunction, which frequently accompanies hearing loss, and multisensory integration changes that occur at the central level. Considering the close anatomical and functional relationship between the vestibular and auditory systems, the reduced effective use of vestibular input in individuals with hearing loss is consistent with the literature [16, 17]. While our exclusion criteria aimed to eliminate overt vestibular disorders, it is important to acknowledge that subclinical vestibular hypofunction often coexists with sensorineural hearing loss due to the anatomical proximity of the cochlea and vestibular organs. Since objective vestibular tests such as vHIT or VEMPs were not performed in this study, the observed reductions in VEST and COMP scores should be interpreted as a global multisensory finding rather than a purely central or purely peripheral deficit. When the present findings are compared with our previous study in cochlear implant users, both groups demonstrated reduced VIS, VEST, and composite posturography scores, indicating impaired multisensory balance integration. However, the pattern of sensory processing differences differed between groups. Cochlear implant users primarily showed increased low registration, sensory sensitivity, and sensory avoidance, whereas hearing-aid users in the present study demonstrated reduced sensory seeking, taste/smell processing, and activity level. This difference may be explained by the presence of surgically induced vestibular disturbance and abrupt sensory reorganization in cochlear implant users, whereas hearing-aid users experience more gradual sensory adaptation associated with chronic sensorineural hearing loss. Therefore, the present study contributes new evidence by demonstrating that even without surgical vestibular insult, hearing loss alone may lead to altered sensation seeking, reduced environmental engagement, and changes in multisensory postural control.

Correlation analysis revealed weak-to-moderate positive associations between subjective sensory processing patterns and objective postural control measures. Specifically, the relationship between Sensation Seeking and VEST performance (ρ=0.382) suggests a potential trend where individuals with higher sensory-seeking tendencies might utilize vestibular inputs more effectively. However, given the moderate strength of these correlations, these findings should be viewed as preliminary observations rather than definitive mechanistic links. According to these approaches, the vestibular system is used more effectively in individuals with high movement and sensory input seeking, and this positively reflects on postural control performance [32, 33]. This situation is thought to result in higher vestibular balance scores in posturography. One of our most striking findings is the negative relationship between the Touch Process and SOM scores. Increased tactile sensitivity or processing issues in this area may weaken an individual’s ability to integrate proprioceptive and tactile inputs into balance strategies. This confirms the “postural instability” observed in individuals with sensory integration disorders through the SOM score, an objective posturography parameter. Furthermore, the fact that participants with high Activity Levels have better VEST and PREF scores indicates that physical activity develops not only muscle strength but also the central nervous system’s ability to distinguish and organize different sensory inputs. Hearing loss, particularly bilateral hearing loss, has been widely reported in the literature to have significant and multidimensional negative effects on individuals’ quality of life [34, 35]. In our study, it was found that the subscale scores for Social Functioning and Physical Functioning on the SF-36 quality of life scale were statistically significantly lower in individuals with hearing loss compared to individuals with normal hearing. In a study conducted on cochlear implant users, cochlear implant users were compared with individuals with normal hearing, and significant differences were found in the areas of mental health and social functioning [10]. Hearing and balance problems can lead to social isolation and a decrease in quality of life by making it difficult to process sensory stimuli adequately [10]. This is consistent with the decline in social functioning and physical function scores observed in our study.

Although there are numerous studies in the literature that address sensory processing, balance, or quality of life separately in individuals with hearing loss, studies that examine the relationship between these areas using a holistic approach are limited. However, it is known that maintaining postural stability depends on the effective integration of vestibular, visual, and somatosensory inputs, and that changes in these processes can directly affect an individual’s participation in daily life activities and quality of life. In this study, the combined assessment of sensory processing profiles, postural stability parameters, and health-related quality of life measures has enabled a more comprehensive understanding of the functional outcomes observed in hearing aid users. The findings suggest that sensory and postural changes accompanying hearing loss may affect not only balance performance but also the individual’s physical, social, and psychosocial functioning. This holistic approach emphasizes the importance of a multidisciplinary perspective in assessment and rehabilitation processes and points to the need for more comprehensive clinical assessment strategies in hearing aid users. Unlike cochlear implant studies, the present study demonstrates that multisensory and postural alterations are not limited to surgically treated hearing loss but are also present in hearing-aid users. This finding highlights the importance of evaluating sensory processing and balance even in individuals using conventional amplification and suggests that rehabilitation strategies should address multisensory integration rather than focusing solely on auditory function.

## Limitations of the study

This study has several limitations. The cross-sectional design limits causal inferences. The self-report nature of the sensory processing assessment and the single-center sample may reduce the generalizability of the findings. Additionally, the use of convenience sampling and the implementation of strict exclusion criteria (e.g., excluding patients with known vertigo or polypharmacy) were necessary for internal validity; however, these choices limit the generalizability of our findings to the broader, often more medically complex, elderly hearing-impaired population. Although age was included as a covariate in our ANCOVA models to mitigate its confounding influence, the complex interplay between hearing loss, aging, and denervation of the sensory systems cannot be entirely decoupled. The observed differences in postural stability may partly reflect shared degenerative processes affecting both auditory and vestibular pathways, rather than a direct isolated effect of hearing loss alone. Furthermore, our study did not employ objective vestibular-specific assessments (e.g., vHIT or VEMPs), which limits our ability to distinguish between central multisensory integration deficits and peripheral subclinical vestibular hypofunction. In addition, multiple statistical comparisons were performed across several outcome domains, which may increase the risk of type I error despite false discovery rate correction. Finally, objective vestibular assessments beyond computerized dynamic posturography (e.g., caloric testing, video head impulse test, or vestibular evoked myogenic potentials) were not performed, limiting detailed pathophysiological interpretation. Future longitudinal and multicenter studies incorporating comprehensive vestibular assessment are needed to clarify causal mechanisms and improve clinical generalizability.

## Conclusion

This study has revealed that sensory processing, postural stability, and quality of life differ in adult hearing aid users compared to individuals with normal hearing. Beyond auditory dysfunction, the findings highlight measurable alterations in multisensory balance control and functional participation in hearing-aid users. Accordingly, considering sensory processing and postural stability together in the clinical assessment of hearing aid users may contribute to the development of more comprehensive approaches.
